# Exploring phosphatidylinositol 5-phosphate 4-kinase function

**DOI:** 10.1016/j.jbior.2014.09.007

**Published:** 2015-01

**Authors:** Simon J. Bulley, Jonathan H. Clarke, Alaa Droubi, Maria-Luisa Giudici, Robin F. Irvine

**Affiliations:** Department of Pharmacology, Tennis Court Road, Cambridge, CB2 1PD, UK

**Keywords:** Phosphatidylinositol, Phosphatidylinositol 5-phosphate, Phosphatidylinositol 5-Phosphate4-kinase, Kinase, Inositide

## Abstract

The family of phosphatidylinositol 5-phosphate 4-kinases (PI5P4Ks) is emerging from a comparative backwater in inositide signalling into the mainstream, as is their substrate, phosphatidylinositol 5-phosphate (PI5P). Here we review some of the key questions about the PI5P4Ks, their localisation, interaction, and regulation and also we summarise our current understanding of how PI5P is synthesised and what its cellular functions might be. Finally, some of the evidence for the involvement of PI5P4Ks in pathology is discussed.

## Introduction

The phosphatidylinositol 5-phosphate 4-kinases (PI5P4Ks, EC 2.7.1.149) are a family of three in most vertebrates – see (Clarke and Irvine, 2012, 2013). It is generally accepted that the reaction they catalyse *in vivo* is the 4-phosphorylation of PI5P, and because of the much lower abundance of this lipid compared to PI4P (the major precursor of PI(4,5)P_2_ and substrate of the phosphatidylinositol 4-phosphate 5-kinases, PI4P5Ks), it is also accepted that their most likely function is to remove PI5P and thus control its levels in the cell. If the pool of PI(4,5)P_2_ synthesised by the PI5P4K route does have a function, it must be a specific and localised one, given that the amount will be much lower than the overall cellular levels of PI(4,5)P_2._ That concept cannot be ruled out, as, for example, PI5P4Ks can be localised to intracellular membranes (e.g. PI5P4Kγ (Clarke et al., 2008)), where PI(4,5)P_2_ is present at very low levels. Here we review some of the current concepts about the PI5P4K family, how their substrate, PI5P, is synthesised in cells, and what the functions of PI5P4Ks and PI5P may be in cellular physiology and pathology.

## Activity, localization and interrelationship of PI5P4Ks

### PI5P4Kγ

It is clear that, at least as assayed *in vitro*, the three isoforms of PI5P4Ks have very different PI5P 4-kinase activities (Bultsma et al., 2010; Clarke and Irvine, 2013; Wang et al., 2010), with several orders of magnitude separating the most active (PI5P4Kα) from the least active (PI5P4Kγ). Indeed, the activity of PI5P4Kγ is so low that we have raised the issue of whether it might not function as a PI5P 4-kinase *in vivo* at all (Clarke and Irvine, 2013). But in its vesicular location, exposed perhaps to a local PI5P pool, with millimolar levels of ATP to drive it at a maximum rate, it may have sufficient activity to impact on local PI5P or PI(4,5)P_2_ levels. Also, we do not yet know for certain how the dimerization of PI5P4Ks with each other (Bultsma et al., 2010; Clarke and Irvine, 2013; Wang et al., 2010) may impact on their activity. When PI5P4Kα is co-immunoprecipitated with overexpressed active or inactive PI5P4Kβ the lipid kinase activity of the complex with active PI5P4Kβ is greater than that of the complex with inactive PI5P4Kβ, and the activity of PI5P4Kβ assayed *in vitro* cannot account for this difference (Bultsma et al., 2010). The implication therefore is that either active PI5P4Kβ is required for full activation of PI5P4Kα, or that PI5P4Kα can activate PI5P4Kβ, and either way, if this applies also to PI5P4Kγ then it may in be more active *in vivo* (as a heterodimer) than *in vitro* assays would suggest.

The idea of PI5P4Kγ as an active PI5P 4-kinase may receive some support from our recent exploitation of a highly specific PI5P4Kγ inhibitor that apparently interacts with the PI5P binding site (J.H.C., M-L.G. et al., unpublished observations). This has clear effects on cell trafficking events in mpkCCD cells, which are mimicked by RNAi knock-down of PI5P4Kγ, and either this is because it is inhibiting the PI5P 4-kinase activity of PI5P4Kγ, or it is displacing the enzyme from its localization in intracellular vesicles and thus altering its ability to target other proteins to these vesicles. In the latter context, as noted above, PI5P4K isoforms can heterodimerise (Bultsma et al., 2010; Clarke and Irvine, 2013; Wang et al., 2010), plus we have produced evidence that PI5P4Kα has the ability to interact with any one of the three PI4P5K isoforms (Hinchliffe et al., 2002), so the concept of PI5P4Kγ being a ‘targeting’ protein is entirely plausible. The same may be true of PI5P4Kβ (discussed below), and the functional consequences of the PI5P4Ks abilities to heterodimerise remains one of the key unknowns about them.

### PI5P4Ks α and β

The interrelationship of the α and β isoforms of PI5P4K is an interesting one that we and others have explored quite extensively, particularly with regard to their localization (and see above for activity considerations). In overexpression experiments, PI5P4Kα tagged at the N- or C-terminus with GFP is largely cytoplasmic when transfected into HeLa cells or cultured rat hippocampal neurons (Ciruela et al., 2000) or DT40 cells (Richardson et al., 2007). The same result was found with Myc-tagged PI5P4Kα in HeLa cells (Bultsma et al., 2010; Bunce et al., 2008) and FLAG-tagged PI5P4Kα in Cos-7 and PAE cells (Hinchliffe et al., 2002). An exception to this pattern is that Boronenkov et al. (Boronenkov et al., 1998) found the FLAG-tagged enzyme to be predominantly nuclear, and sometimes associated with nuclear speckles, in cultured fibroblasts.

Most of the above studies also examined the localization of overexpressed PI5P4Kβ. Ciruela et al. (2000) found it to be predominantly nuclear when N- or C-terminally tagged with GFP in HeLa cells or cultured rat hippocampal neurons. In contrast Bunce et al. (2008) reported 80% of Myc-tagged PI5P4Kβ to be cytoplasmic in HeLa cells, and Bultsma et al. (2010) also found PI5P4Kβ to be predominantly cytoplasmic in HeLa cells, in this case detected by an HA tag. Interestingly, in both of these papers some of the minority of PI5P4Kβ seen in the nucleus was in nuclear speckles (Bultsma et al., 2010; Bunce et al., 2008), and Bunce et al. (2008) demonstrated that this pool of enzyme co-localized with SPOP. Co-localization with nuclear speckles was also reported with FLAG-tagged PI5P4Kβ in cultured fibroblasts but in this case the majority of PI5P4Kβ was nuclear (Boronenkov et al., 1998). In DT40 cells GFP-tagged PI5P4Kβ is largely nuclear upon overexpression (Richardson et al., 2007).

Given these confusing results from overexpression it is perhaps not surprising that studies of the endogenous PI5P4Ks are also in turn at odds with them. Boronenkov et al. (1998) found, using an isoform-specific antibody, that most PI5P4Kα is nuclear and that some of the staining co-localizes with nuclear speckles; to date this is the only paper to date suggesting a predominant nuclear localization of endogenous PI5P4Kα. Bultsma et al. (2010) addressed the localization of the endogenous enzymes by fractionating cytoplasm from nucleus in HEK-293 cells and then immunoprecipitating and blotting with isoform-specific antibodies. The great majority of both PI5P4Kα and PI5P4Kβ was cytoplasmic, and a significant amount of the cytoplasmic PI5P4Kβ was membrane-bound.

The other studies to examine the localization of the endogenous enzymes have been our studies employing genomic epitope-tagging in DT40 cells. Richardson et al. (2007) endogenously FLAG-tagged PI5P4Kβ and found it to be almost exclusively nuclear. This result was confirmed in a subsequent study with an endogenous FLAG-hexaHis tag (Wang et al., 2010). In the latter study a separate cell line with PI5P4Kα genomically tagged showed that 40% of PI5P4Kα was nuclear. It is also interesting that the 60% that was cytoplasmic was exclusively membrane-bound rather than cytosolic, although to which membrane or membranes was unclear.

This overall confusing and apparently contradictory picture may now be partly on the way to a resolution from some of our recent observations in DT40 cells (which, being derived from birds, have no PI5P4Kγ to confuse the picture (Clarke and Irvine, 2013)). Firstly, the concept that PI5P4Kβ can target PI5P4Kα to the nucleus via heterodimerization (Bultsma et al., 2010; Wang et al., 2010) gains a different perspective from our discovery that the heterodimerization may be dynamic (i.e. with an exchange of subunits over a time course of a few minutes, J.H.C. et al., unpublished observations), opening up the idea of PI5P4Kβ ‘shuttling’ PI5P4Kα in and out of the nucleus. On top of this is our recent discovery (S.J.B., A.D., et al., unpublished observations) that the nuclear localisation of PI5P4Kβ is itself dynamic and regulated, as it can vary from almost 90% cytosolic to 90% nuclear, depending on the growth state of the cells. Thus it is possible to imagine circumstances where almost any combination of nuclear and cytoplasmic localisation of PI5P4Ks α and β could be observed. This is, essentially, what is described above.

## Route and regulation of PI5P synthesis

If we accept that a major function of PI5P4Ks is to remove PI5P and thus regulate its levels, then how PI5P is synthesised, and where, becomes an imporant question in our quest to understand the physiology of these enzymes. From a current perspective, PI5P synthesis is best classified as being either PIKfyve-dependent or PIKfyve-independent. PIKfyve-dependent synthesis may occur in one of two ways: either by direct phosphorylation of PI to PI5P; or by PIKfyve-mediated phosphorylation of PI3P to PI(3,5)P_2_ which is then dephosphorylated by a 3′-phosphatase to PI5P. PIKfyve-independent synthesis of PI5P is most likely to progress by the action of a 4′-phosphatase on PtdIns(4,5)P2. These possible routes of synthesis are not mutually exclusive and the balance between them may depend upon the cellular compartment and the physiological or pathological context. As a background to this discussion we should note that across a range of cell lines the basal level of PtdIns5P is 0.5–2% of that of PtdIns4P (Roberts et al., 2005; Sarkes and Rameh, 2010).

### PIKfyve-dependent synthesis of PI5P

Human PIKfyve co-localizes predominantly with the early endosomal marker EEA1, although some PIKfyve also segregates into a later endosomal compartment (Cabezas et al., 2006). Sbrissa et al. (1999) demonstrated that native or recombinant PIKfyve can synthesize PI5P from PI as well as synthesizing PI(3,5)P_2_ from PI3P *in vitro*. Zolov et al. (2012) addressed the *in vivo* contribution of PIKfyve to PI5P and PI(3,5)P_2_ using fibroblasts from a PIKfyve gene-trap mouse. In homozygous gene-trap fibroblasts PIKfyve is reduced to about 10% of the wild-type level and can be reduced to undetectably low levels by shRNA in the gene-trap fibroblasts. In the homozygous gene-trap fibroblasts PI(3,5)P_2_ and PI5P are reduced to 50% of wild-type levels. In shRNA expressing cells PI(3,5)P_2_ is rendered undetectable, PI3P is increased fivefold, and PI5P is reduced to 15% of the wild-type level (Zolov et al., 2012). These results provide evidence that in these cells most of the cellular pool of PI5P is dependent upon PIKfyve, but the remaining 15% is presumably synthesized via a PIKfyve-independent route. Intriguingly, the level of PI(4,5)P_2_ was reduced by about one third following shRNA treatment of homozygous gene-trap fibroblasts. This is in agreement with data demonstrating a 20% reduction in PI(4,5)P_2_ in NIH3T3 cells following treatment with the PIKfyve inhibitor YM201636 (Jefferies et al., 2008), and raises the possibility that the PIKfyve-dependent pool of PtdIns5P is in some way contributing to, or regulating the synthesis of, the cellular pool of PtdIns(4,5)P_2_ (Zolov et al., 2012).

ßAs discussed above, the dependence of the bulk of PI5P upon PIKfyve could be due either to D5 phosphorylation of PI by PIKfyve, or due to the immediate precursor of PI5P being PI(3,5)P_2_, which is dephosphorylated by a 3′-phosphatase. Zolov et al. (2012) sought to distinguish between these alternatives in three ways. Firstly, PIKfyve was inhibited in wild-type fibroblasts with the PIKfyve inhibitor YM201636 (Jefferies et al., 2008). PI(3,5)P_2_ levels fell with a half life of substantially less than 2.5 min, whilst the half life of PI5P was 4.5 min. Secondly, nutrient re-feeding was used to stimulate PIKfyve activity (Bridges et al., 2012) and resulted in PI(3,5)P_2_ and PI5P returning to pre-starvation levels in 2.5 and 5 min respectively, suggestive of a precursor-product relationship between these phospholipids (Zolov et al., 2012). Thirdly, the authors repeated the experiments of McEwen et al. (McEwen et al., 1999) and confirmed that PIKfyve has little or no ability to synthesize PI5P in *S. cerevisiae,* an organism that synthesizes very little PtdIns5P. Together these data suggest that most (about 85%) of the cellular PI5P in mouse fibroblasts is dependent upon PIKfyve, and that PIKfyve generates PI(3,5)P_2_ which is in turn subject to the activity of a 3′-phosphatase, the most likely candidate for which is a myotubularin (Zolov et al., 2012). Strangely for an enzyme/product relationship, PI5P is capable of stimulating the activity of a number of myotubularins thus forming a positive feedback loop (Schaletzky et al., 2003).

Oppelt et al. (2013) provided direct evidence of the involvement of a myotubularin, MTMR3, in PI5P production in human fibroblasts. In this study knockdown of VPS34, the catalytic subunit of PI 3-kinase III, reduced cell migration presumably due to decreased PI3P production or a downstream consequence thereof. Cell migration could be rescued by exogenous addition of PI5P but not other inositol lipid species. A siRNA screen for PI3P effectors involved in cell migration generated hits on, amongst others, PIKfyve and the phosphoinositide 3′-phosphatase MTMR3 (Oppelt et al., 2013). As these two enzymes can act in concert to generate PI5P from PI3P via PI(3,5)P_2_ (above), Oppelt et al. (2013) provided convincing evidence that the pathway to the pool of PI5P important in their migration phenotype is indeed via PI(3,5)P_2_.

Interestingly Oppelt et al. (2013) found that while knockdown of PIKfyve or MTMR3 decreased PI5P and migration in cells stimulated to migrate with FGF1, no statistically significant PI5P decrease occurred in unstimulated cells. These unstimulated cells nevertheless migrated less efficiently. Perhaps PI5P is produced via different routes in stimulated and unstimulated cells, or perhaps any decrease in PI5P is more difficult to measure against the much lower baseline in unstimulated cells. It is worth noting in this context that whereas transfecting PI5P4Kα into HeLa cells had no effect on resting PI5P levels (Roberts et al., 2005), transfection did attenuate the increased PI5P production in cells induced by tyrosine kinase inhibition (Wilcox and Hinchliffe, 2008). In support of the role of myotubularins in PI5P production, primary fibroblasts from Mtmr2 knockout mice have about 20% of the wild-type level of PtdIns5P and 150% of the wild-type level of PI(3,5)P_2_ as measured by mass assay and HPLC respectively (Vaccari et al., 2011). In summary, the majority of data currently available suggests that the PIKfyve-dependent pool of PI5P is generated via PI(3,5)P_2_ and a myotubularin rather than by direct phosphorylation of PI.

There are some exceptions to this, however. In BTC6 cells [^3^H]inositol incorporates into PtdIns5P much faster than it incorporates into PI4P, PI(4,5)P_2_ and PI(3,5)P_2_ (Sarkes and Rameh, 2010). This is not the case in HeLa cells, suggesting that in some cells direct phosphorylation of PI to PI5P may be an important route of synthesis (Sarkes and Rameh, 2010). In another study YM201636 used at low doses was found preferentially to inhibit the generation of PI5P compared to PI(3,5)P_2_ both *in vitro* and *in vivo* (Sbrissa et al., 2012). This low dose selectivity was used to probe phenotypes dependent upon PI5P such as insulin-induced stress fibre disassembly and GLUT4 translocation. The results suggested that the PI5P pool important for mediating these events was depleted at low doses of YM201636; given the preferential depletion of PI5P compared to PI(3,5)P_2_ at the relevant inhibitor dose this PI5P pool was suggested to be generated directly from PI rather than via PI(3,5)P_2_ (Sbrissa et al., 2012).

### PIKfyve-independent synthesis of PI5P

The only known PIKfyve-independent route of PI5P synthesis for which *in vivo* evidence exists is the hydrolysis of PI(4,5)P_2_ to PI5P by the phosphoinositide 4-phosphatases. By homology to the bacterial PI(4,5)P_2_ 4-phosphatase IpgD (Niebuhr et al., 2002) active site motif, two mammalian PI(4,5)P_2_ 4-phosphatases were discovered, now called type I and type II (Ungewickell et al., 2005; Zou et al., 2007). Overexpression of the type I isoform depletes PI(4,5)P_2_ and increases basal PI5P (Ungewickell et al., 2005; Zou et al., 2007), but the type II isoform has to date only been demonstrated to possess 4-phosphatase activity *in vitro* (Ungewickell et al., 2005). In senescent cells the type I and type II 4-phosphatases co-localize with late endosomal and lysosomal markers, whilst under conditions of pro-apoptotic stress the type I isoform translocates to the nucleus (Zou et al., 2007).

Given that the enzymes thought to be responsible for PI5P synthesis are localized to intracellular compartments and/or the nucleus, it is an intriguing question as to how the bulk of PtdIns5P, which in a resting cell is probably in the plasma membrane (Roberts et al., 2005; Sarkes and Rameh, 2010), is synthesized. It could, of course, be made in intracellular compartments and trafficked to the plasma membrane, or another route of synthesis may be present at the plasma membrane. The PI4P5Ks are able to produce PI5P *in vitro* by direct phosphorylation of PI (Tolias et al., 1998), though there is no *in vivo* data to support PI5P synthesis by the PI4P5ks. As a sepeculative thought, there is now good evidence that PI 4-kinase type III α is localized to the plasma membrane rather than to the ER as previously believed (Nakatsu et al., 2012; Wu et al., 2014). Were PI 4-kinase type III α to act as a PI 5-kinase rather than a 4-kinase only a few percent of the time it could easily account for the generation of all the PI5P at the plasma membrane. It is also worth adding that it is known that some PI(4,5,P)_2_ 5-phosphatases can, again at a few percent, remove the 4-phosphate instead of the 5, and thus could serve as a source of PI5P in the plasma membrane.

## Nuclear functions of PI5P

The first PI5P-interacting protein to be described was the chromatin-associated protein ING2 (Gozani et al., 2003). Although the PHD finger of ING2 is able to bind phosphoinositides other than PI5P, the interaction with PI5P is the most convincing and reproducible across a range of assays both *in vitro* and *in vivo* (Gozani et al., 2003). ING2 is a tumour suppressor that is critical for p53 acetylation and subsequent p53-mediated cell death in response to DNA damage. Overexpression of PI5P4Kβ to deplete PI5P alters the subcellular localization of ING2 and inhibits p53-mediated cell death (Gozani et al., 2003). Similarly, overexpression of the PHD finger of ING2 to sequester PI5P abrogates the ability of ING2 to activate p53-mediated apoptosis. More recently PI5P binding to ING2 was shown to drive ING2 promoter occupancy and subsequent gene repression at a subset of ING2 target genes (Bua et al., 2013). These findings are consistent with a model in which PI5P stabilizes ING2 at discrete genomic sites (Bua et al., 2013). It is worth noting that we found that nuclear PI5P levels do fluctuate throughout the cell cycle (Clarke et al., 2001) but the functional consequences of this are unknown.

PI5P can also regulate gene transcription in plants by exerting effects on histone modification. ATX1 is a plant histone methyltransferase homologous to the mammalian trithorax proteins and responsible for transcriptional regulation to control plant development and environmental stress responses, which preferentially binds PI5P *in vitro*, and addition of exogenous PI5P to cells negatively regulates the function of ATX1 as assayed by whole genome expression profiles (Alvarez-Venegas et al., 2006). Exogenous PI5P also leads to a shift of ATX1 from the nuclear to cytoplasmic compartment (Alvarez-Venegas et al., 2006). In a separate study, drought stress in Arabidopsis was shown to increase cellular PI5P levels, re-localize ATX1 from the nucleus to the cytoplasm, and reduce the transcription of an ATX1 target gene (Ndamukong et al., 2010). Interestingly, the pool of PI5P generated in response to stress in Arabidopsis, but not the basal pool, seems to be dependent upon a plant myotubularin homologue AtMTM1 (Ndamukong et al., 2010).

Given that the nuclear effectors of PI5P seem mostly to be involved in stress responses the mechanisms of nuclear PI5P regulation under conditions of stress is an important question. It is now clear that, at least in some circumstances, PI5P4Kβ and p38-MAPK are regulators of this pool of lipid. PI5P4Kβ can be nuclear (see above) and in response to UV irradiation PI5P4Kβ is directly phosphorylated by p38-MAPK (Jones et al., 2006). This phosphorylation event decreases the PI5P4Kβ activity associated with PI5P4Kβ immunoprecipitates and nuclear PtdIns5P levels increase (Jones et al., 2006). At least *in vitro,* however, PI5P4Kβ has very little activity compared to PI5P4Kα (see above), which therefore raises the question as to how the relatively inactive PI5P4Kβ could contribute meaningfully to PI5P removal. However, the heterodimerisation between the PI5P4K isoforms discussed above may resolve this possible conundrum and there is some indirect evidence that p38-MAPK can also phosphorylate PI5P4Kα (see below, (Keune et al., 2012)), so perhaps the important target of p38-MAPK is actually PI5P4Kα. Alternatively, phosphorylation of PI5P4Kβ could reduce the kinase activity associated with PI5P4Kβ immunoprecipitates by modulating the interaction between the two isoforms as discussed above.

UV irradiation is not the only stress capable of causing an increase in nuclear PI5P. Oxidative stress induced by hydrogen peroxide can do the same, except in this case the PI5P generated appears to be largely independent of the p38-MAPK/PI5P4Kβ pathway (Jones et al., 2013; Keune et al., 2013a). Both PI5P4Ks α and β interact with the prolyl isomerase Pin1 *in vivo* upon cellular exposure to oxidative stress (Keune et al., 2012, 2013a). This interaction inhibits the catalytic activity of both PI5P4K isoforms *in vitro,* and leads to enhanced PtdIns5P production in response to oxidative stress *in vivo* (Keune et al., 2012, 2013a). The Pin1/PI5P4K interactions are dependent upon p38-MAPK, and although there is no direct evidence that p38-MAPK phosphorylates PI5P4Kα this seems to be the most likely explanation given that the p38-MAPK phosphorylation site is conserved between PI5P4Ks α and β. PI5P generation is suggested to be protective against oxidative stress but contradictorily, Pin1 null MEFs are resistant to oxidative stress and generate more PI5P in response to such stress than their wild type counterparts (Jones et al., 2013; Keune et al., 2012, 2013a). Clearly there is more to be learned in the complex interactions between PI5P4Ks and stress.

The p38-MAPK/PI5P4K pathway is also able to regulate the ubiquitination of nuclear proteins. A yeast two-hybrid screen identified an interaction between PI5P4Kβ and SPOP (Bunce et al., 2008). SPOP is a protein that recruits substrates, including PI5P4Kβ, to Cul3-based ubiquitin ligases (Bunce et al., 2008). Elevation of PI5P by overexpression of either PI(4,5)P_2_ 4-phosphatases or kinase dead PI5P4Kβ results in increased activity of the Cul3-SPOP ubiquitin ligase, and this increased activity can be prevented by an inhibitor of the p38-MAPK pathway (Bunce et al., 2008). These findings suggest a positive feedback loop whereby cell stress activates p38-MAPK, which both promotes Cul3-SPOP activity and inhibits PI5P4Kβ through direct phosphorylation. The latter effect leads to an increase in nuclear PI5P, which in turn promotes p38-MAPK activity leading to further activation of Cul3-SPOP. The targets of Cul3-SPOP include PI5P4Kβ, and degradation of this PI5P4K leads to further elevation of nuclear PI5P (Bunce et al., 2008).

Pathogens can also impinge upon nuclear PI5P signalling. Viral infection appears to stimulate PI5P production via a PIKfyve-dependent pathway, and stimulates the phosphorylation and activation of the transcription factor IRF3 by its kinase TBK1 (Kawasaki et al., 2013). This event induces interferon production. Both IRF3 and TBK1 can bind PI5P *in vitro,* and a model in which PI5P correctly localizes the two proteins for phosphorylation has been suggested (Kawasaki et al., 2013).

Finally, a recent report extends the nuclear functions of PI5P to include regulation of UHRF1, a multi-domain protein that links histone modification states to DNA methylation (Gelato et al., 2014). PI5P binds with a clear specificity to a polybasic region in the C-terminus of UHRF1, resulting in a conformational change in the protein thus allowing its tandem tudor domain to interact with Histone 3 trimethylated on lysine 9. Gelato et al. (2014) also provide evidence that PI5P is indeed the endogenous factor inducing this change in nuclear extracts.

## Cytoplasmic functions of PI5P

Compared to the growing number of well-defined (in both molecular and cellular terms) functions for PI5P in the nucleus, those in the cytoplasm are much less clear. As yet there are no cytoplasmic proteins of sufficient PI5P binding specificity to be classed as convincing PI5P effectors, especially given the relatively higher levels of PI3P and (especially) PI4P in cells (see, e.g. (Roberts et al., 2005)). Moreover, many of the investigative trails towards cytoplasmic PI5P functions involve either: (a) knockout mice (Carricaburu et al., 2003; Emerling et al., 2013; Lamia et al., 2004), with the inevitable compromise that insights into acute signalling pathways that these generate are indirect and may be the result of compensatory developmental events, or (b) the employment of bacterial PI(4.5)P_2_ 4-phosphatases to generate PI5P (see (Pendaries et al., 2006; Ramel et al., 2009, 2011) and (Payrastre et al., 2012) for review), where the amount of PI5P generated is much higher than those that exist endogenously, so drawing parallels between pathology and physiology can be difficult.

### PI5P and Akt

Nevertheless, a well defined role for extra-nuclear PI5P at the plasma membrane where it promotes Akt activation following infection by *Shigella flexneri* (Pendaries et al., 2006), and the possible mechanisms and consequences of this have been reviewed by Payrastre et al. (2012), and importantly, support for a role of PI5P in promoting Akt signalling comes additionally from a number of indirect sources that do not rely on bacterial toxins or knock-out mice. Firstly, PIKfyve overexpression in CHO-T cells increases insulin-stimulated Akt phosphorylation at Ser473 but not at Thr308 (Ikonomov et al., 2002). Secondly, overexpression of PI5P4Kβ in CHO cells stably overexpressing the human insulin receptor resulted in attenuation of overexpressed HA-Akt phosphorylation at the Thr308 site (Carricaburu et al., 2003). Thirdly, acute PI5P4Kα knockdown in THP1 AML cells increases cellular PI5P and increases Akt phosphorylation at Ser473 (Jude et al., 2014).

The increased Akt phosphorylation in this latter study was proposed to be due to upregulation of mTOR, although there was no direct evidence that PI5P is the crucial mediator (Jude et al., 2014); having said that, Grainger et al. reported that exogenous PI5P could increase Akt phosphorylation in unstimulated myotubes (Grainger et al., 2011). It is important to note that PI5P (and PI(4,5)P_2_) have been reported to allosterically activate PTEN (Campbell et al., 2003; Redfern et al., 2008), PI5P can inhibit SHIP2 (Pendaries et al., 2006) and activate the myotubularins (Schaletzky et al., 2003) and Akt phosphatases (Ramel et al., 2009) to name but a few possible regulatory influences of this lipid. Moreover, our recent studies on DT40 cells in which we can remove PI5P4Ks chronically by knock-out or acutely by genomically tagging them with the auxin degron (Nishimura et al., 2009), have exposed several complex links between PI5P4Ks and Akt signalling that differ between chronic and acute removal (A.D., S.J.B. et al. unpublished data). Moreover, by mutating the single remaining allele of PI5P4Kα in a DT40 line where the other alleles have been deleted, we have found evidence that at least one function of this enzyme may indeed be to synthesise PI(4,5)P_2_, rather than just remove PI5P. Overall this is a complex relationship that will need more experimentation to tease out.

### PI5P and the actin cytoskeleton

Although the molecular target is not yet defined, a very convincing story associating PIKfyve-generated PI5P with the regulation of the actin skeleton in cell migration has emerged from a number of laboratories. Earlier work using the *Shigella* phosphatase IpgD had pointed to PI5P and actin reorganisation being connected (Niebuhr et al., 2002). Expression of recombinant IpgD in HeLa cells induces the formation of membrane blebs and loss of actin stress fibres, whilst in NIH-3T3 cells cytoskeletal-membrane adhesion is reduced as assayed by tether force microscopy (Niebuhr et al., 2002). These early studies could not distinguish between depletion of PI(4,5)P_2_ or generation of PI5P as the crucial event in IpgD-mediated cytoskeletal re-arrangement, but subsequent studies in different contexts make PI5P the most likely candidate.

Two of the many cellular effects of insulin are to promote F-actin stress fibre disassembly and to induce the trafficking of GLUT4 transporters to the plasma membrane (Ikonomov et al., 2002). Overexpression of the PI5P-generating enzyme PIKfyve or microinjection of PI5P mimics these insulin effects whilst sequestration of PI5P with the probe 3xPHD ING2 inhibits insulin-induced changes in the cytoskeleton and GLUT4 translocation (Sbrissa et al., 2004). Furthermore, when the PIKfyve inhibitor YM201636 is used at a concentration of 160 nM, a concentration that selectively impairs the production of PI5P over PI(3,5)P_2_, insulin-induced F-actin stress fibre disassembly is dramatically reduced (Sbrissa et al., 2012). This suggests that PI5P is required downstream of insulin for stress fibre disassembly and that the crucial pool of PI5P is PIKfyve-dependent and Ikonomov et al., (Ikonomov et al., 2002) found that overexpression of dominant negative PIKfyve mutants in 3T3-L1 adipocytes inhibited insulin-stimulated GLUT4 translocation.

Additional data supporting a link between PI5P and GLUT4 translocation comes from the work of Grainger et al. (Grainger et al., 2011), who showed that transfecting cells with PI4P5Kα or adding exogenous PI5P had opposing effects (inhibiting or increasing respectively) on GLUT4 translocation.

In a recent elegant set of studies from Wesche's lab (Haugsten et al., 2013; Oppelt et al., 2014, 2013), already discussed in the context of routes of PI5P synthesis, the authors have suggested that PI5P is a crucial signalling intermediate in FGF1-stimulated cell migration. FGF1 induces PI5P production and promotes cell migration in BJ fibroblasts (Oppelt et al., 2013); these effects can be attenuated by knockdown of the PI5P-generating enzymes PIKfyve and MTMR3, plus MTMR3 knockdown additionally induces re-arrangements of the actin cytoskeleton (Oppelt et al., 2013). Furthermore, overexpressing PI5P4Kα to deplete cellular PI5P decreases cell migration velocity (Haugsten et al., 2013). More recently Oppelt et al. (2014) have implicated Rac as an effector of these effects, and highlighted the importance of the migration to metastasis.

## PI5P4Ks and pathology

Given the discussions above, it is obvious that there is plenty of scope for the involvement of PI5P4Ks with pathological states. Three recent reports have highlighted directly the potential involvement of PI5P4Ks in pathology, particularly in cancer. Using an array of techniques, in particular knock-out mice, Emerling et al. (2013) in a comprehensive study have drawn a compelling link between PI5P4Ks α and β and cancers that include p53 deficiency as part of their pathology. The relative separate contributions of the two PI5P4Ks to this story is not yet very clear – no doubt that fact that they interact (above) might make this distinction complicated – but the effects of deleting the two enzymes is clear.

A pair of papers from Divecha and collaborators has shown that both α and β PI5P4Ks have their own individual contributions to make to oncogenesis. Keune et al. (2013b) found a correlation between low PI5P4Kβ expression in human breast tumors and reduced patient survival. The mechanism is not yet fully revealed and is clearly complex, involving among other things the regulation of E-cadherin.

In the other study, Jude et al. (2014) showed that in a targeted knock-down screen of inositide-metabolising enzymes, PI5P4Kα emerged with a strong role in acute myeloid leukaemia (AML) cell proliferation and survival. PI5P4Kα was essential for the clonogenic and leukaemia-initiating potential of human AML cells, and for the clonogenic potential of human and murine AML cells. They explored possible mechanisms for these, which include cyclin-dependent kinase inhibitory proteins, and perhaps most enticingly, they found that PI5P4Kα may not be required for normal (as compared with transformed) clonogenic or multilineage potential, raising the hope that inhibiting PI5P4Kα might target only cancerous cells. The recent characterisation of a potential PI5P4Kα inhibitor (Davis et al., 2013) can only raise further such hopes for the future therapeutic targeting of PI5P4Ks. It is time that these enzymes emerged into the limelight.
